# Testicular Cell–Conditioned Chitosan Thermosensitive Hydrogel as an Excellent Substrate for Culture and Proliferation of Spermatogonia Cells

**DOI:** 10.1155/bmri/6693200

**Published:** 2025-06-09

**Authors:** Monireh Mahmoodi, Mahdieh Gholipour-Malekabadi

**Affiliations:** Department of Biology, Faculty of Science, Arak University, Arak, Iran

**Keywords:** coculture, extracellular matrix, infertility, Sertoli, spermatogonia cells, tissue engineering

## Abstract

Successful in vitro transplantation of spermatogonia cells (SCs) requires effective culture systems for the proliferation of SCs. The natural extracellular matrix (ECM) creates a suitable microenvironment for stem cell culture. In the present study, a temperature-sensitive hydrogel scaffold was fabricated from chitosan (TCTS), a biocompatible polysaccharide with the ability to turn into gel using *β*-glycerophosphate as a crosslinker at 37°C. Then, the surface of the hydrogel scaffold was covered with the ECM secreted from testicular cells (TC-TCTS) to provide an excellent substrate for the adhesion and proliferation of SCs toward sperm-producing cells. The synthesized scaffold had a uniform morphology with high biocompatibility and adhesion for both testicular cells and SCs. SEM micrographs, DNA content, and DAPI confirmed the presence of ECM and successful removal of the TC from the hydrogel after decellularization. TC-TCTS showed a higher rate of water absorption and weight loss (around 80%) compared to TCTS. The survival of SCs seeded on the TC-TCTS hydrogel showed a significant increase compared with TCTS. The SCs cultured on TC-TCTS were much more elongated and had a greater tendency to stick to the surface of the hydrogel compared with TC-TCTS. Molecular studies showed a significant increase in the expression of proliferation-associated genes (around 4.5-fold for *id4* and around 7-fold for *plzf*) in SCs cultured on the TC-TCTS compared with TCTS and control. Our findings confirmed that the TC-TCTS can provide an excellent microenvironment biomimicking the natural ECM of seminiferous tubules for adhesion and proliferation of SCs and possible production of sperm in vitro in the future investigations.

## 1. Introduction

Among the 15% of infertility problems in couples, half of them are related to men [1]. According to a report established in 2020, spermatogenesis defects are diagnosed in half of the cases of infertility disorders diagnosed in men [2]. Although many efforts have been made to treat such disabilities, the results are not satisfactory [3–6]. Today, with the increase of infertility and the decrease of gamete donors, finding a method to produce a source of gametes for research and treatment of infertility is very valuable [7]. Studies on in vitro culture systems are tried to provide the perspective of fertility preservation. Various methods focusing on testicular cell (TC) culture, tissue culture, and coculture systems are developed to provide differentiation of male germ cells [8–10]. In recent years, the differentiation of stem cells such as embryonic stem cells and adult stem cells toward germline cells in both two-dimensional (2D) and three-dimensional (3D) cultures and the possible production of functional sperm in vitro has introduced a new treatment strategy to this challenge [11–13]. Spermatogenesis, as a very complex process, is controlled by various autocrine, endocrine, and paracrine and direct contact signaling (cell–cell or cell–extracellular matrix (ECM)) [14–18]. Since the behavior of cells in 2D and 3D culture environments is completely different, recently the use of 3D tissue engineering scaffolds in the proliferation and differentiation of stem cells into sperm-producing cells has attracted the attention of researchers in this field. Tissue engineering provides a microenvironment for cells that induces the differentiation of stem cells in a targeted manner with the maximum ability to mimic the target tissue [19–22]. Cell scaffolds provide a 3D microenvironment (in most cases) for the cell and give the cell a feeling of being in a natural tissue. The placement of cells (especially stem cells) in the 3D environment has a great impact on their behavior, similar to the inside of the body, while stem cells show a completely different behavior in the 2D environment [23, 24]. Many studies have been done to make suitable scaffolds to mimic natural tissue. A suitable scaffold should be biocompatible, should have appropriate mechanical properties and porosity, and should not be toxic to cells [25–29]. In recent years, ECM has been proposed as an attractive biological material in biomedicine. ECM provides many biological signals for cell migration, proliferation, guidance, and promotion of differentiation [30] and is widely used as biological biomaterials for tissue engineering purposes [31–37].

In the present study, in order to increase the amount of mimicry of spermatogenic tubule tissue, a temperature-sensitive hydrogel scaffold was made from chitosan (TCTS), and then, the surface of the hydrogel scaffold was conditioned with the ECM secreted from TCs so that the resulting scaffold can be used as a support for the adhesion and proliferation of spermatogonia cells (SCs) and, as a result, a platform for the proliferation of stem cells toward sperm-producing cells. In this study, to fabricate a hydrogel film on the plastic surface of the cell culture plate, chitosan was crosslinked with an optimized concentration of *β*-glycerophosphate, as a crosslinker, to endow the hydrogel with a thermosensitive property to form gel at 37°C before cell culture. Chitosan, a derivative of chitin with deacetylation and cationic properties, is a well-known biomaterial with excellent biocompatibility, appropriate biodegradability, low toxicity, and antibacterial activity [38–41]. Chitosan has been widely used for the fabrication of tissue engineering scaffolds and substrates for stem cell culture, expansion, and differentiation in various forms of hydrogels, film, sponges, and electrospinning [42]. On the other hand, hydrogels possess unique properties such as a high content of water, biomimicking natural tissue, and excellent porosity which made them an excellent choice for stem cell culture and expansion [43, 44]. Chitosan alone or in combination with other materials has been used for spermatogonia stem cells [45–47]. Until now, the TCTS conditioned with the ECM of TCs for the culture, expansion, and proliferation of SCs with the aim of providing a scaffold imitating the 3D structure and content of the spermatogenic tubes has not been investigated in the laboratory. Considering the ability to localize the present technology as a product in the field of tissue engineering and reproductive biology, as well as the virginity of the field of research in this domain, the present project can bring many innovations and serve as a suitable platform for future research in this field.

## 2. Materials and Methods

### 2.1. Fabrication of Thermosensitive Chitosan Hydrogel (TCTS)

The TCTS was fabricated by a procedure described by Azadbakht et al. [48]. In brief, 2%*w*/*v* CTS was prepared in 0.1 M acetic acid and then placed on the stirrer for 24 h to dissolve the powder well in the solution. In order to make a temperature-sensitive hydrogel scaffold, CTS solution was first placed on the stirrer in a room with a temperature of 4°C, and 40%*w*/*v* beta-glycerol phosphate salt was added dropwise to the solution. This mixing continued until the complete dissolution of the salt in the chitosan solution and reached a uniform solution.

### 2.2. Characterization of TCTS

#### 2.2.1. Gelation Assay

Gelation time of the TCTS was determined by inverted tube assay [49]. Nine glass tubes (*n* = 3) were filled with 3 mL of TCTS at 4°C. The tubes were incubated at 4°C, 25°C, and 37°C for 15 min with an angle of 45°. The tubes were checked for gelation and photographed to confirm the response of the hydrogel to raising temperature.

#### 2.2.2. Morphology Under SEM

The TCTS hydrogels were kept at −80°C for 24 h and then freeze-dried for 24 h to form a 3D network. The samples were sputter coated with gold nanoparticles and viewed under SEM (Philips XL30) at an accelerating voltage of 25 kV [35].

### 2.3. Conditioning of CTS With TCs

#### 2.3.1. Isolation and Culture of TCs

The TCs were isolated from 3 to 6-day-old male mice (NMRI) by a protocol described elsewhere [50]. In this way, tissue samples after washing in phosphate-buffered saline (PBS) solution containing 1% antibiotics were treated with enzymatic digestion solution including Dulbecco's modified Eagle medium (DMEM) containing 1 mg/mL collagenase, 1 mg/mL hyaluronidase, and DNase I 0.05 mg/mL for 15 min at 37°C. Then, in order to remove the interstitial tissue, small pieces of tissue were centrifuged, and after discarding the supernatant, the resulting cell sediment was washed with PBS. In the second stage, the cell sediments were incubated again using the mentioned enzymes. After complete digestion, the samples were centrifuged in the same way. Finally, the isolated cells were incubated for 4 h in a petri dish at 37°C, and after the TCs adhered to the bottom of the dish, the cells were cultured in DMEM culture medium supplemented with 10% fetal bovine serum (FBS) and antibiotics.

#### 2.3.2. Culture of TC on TCTS and Conditioning

The TCTS (1 mL) was poured onto a cell culture plate (20-mm petri dish polystyrene) and incubated at 37°C for 30 min to form a gel. The TCTS-coated petri dishes were sterilized using UV irradiation (30 min). Then, 5 × 10^4^ TCs were cultured on the TCTS and incubated in a DMEM supplemented with 10% FBS and antibiotics for 14 days at 37°C. The medium was gently changed every 3 days. After 14 days, the TC-cultured TCTS sample was gently washed with sterile PBS twice and then placed in a cryotube and placed in a liquid nitrogen tank for 10 min. Immediately, the cryotube containing the scaffolds was placed in PBS at 37°C for 10 min. This step was repeated four times. Finally, the cell–scaffold construct was gently washed again with PBS, incubated at −80°C for 24 h, freeze-dried, and kept at −20°C until use.

### 2.4. Characterization of TC-Conditioned CTS

#### 2.4.1. Morphology Under SEM

The surface of the TC-seeded and conditioned TCTS hydrogels was viewed under SEM. For this purpose, the samples were placed on a special double-sided adhesive, and after being placed in the device, it starts to rotate and at the same time gold particles were sprinkled on the surface of the sample. The surface of the hydrogels was observed under SEM (Philips XL30).

#### 2.4.2. DAPI Staining

DAPI solution (Sigma, United States) was used to stain the nucleus in the scaffolds. The TCTS, TC-seeded, and conditioned TCTS hydrogels were stained with DAPI to confirm the presence of TC in TC-seeded TCTS hydrogels and the removal of the TC from conditioned TCTS hydrogels. The DAPI-stained samples were analyzed using a fluorescence Olympus microscope (BX51, Japan).

#### 2.4.3. DNA Content Assay

DNA was extracted from TC-seeded and conditioned TCTS hydrogels to confirm the successful removal of the TC from TCTS after conditioning. For this aim, DNA was first extracted using a kit (QIAamp DNA Mini Kit, Qiagen, Germany). Then, the amount of extracted DNA was measured using a NanoDrop Spectrophotometer (Thermo Scientific, Venlo, Netherlands) at a wavelength of 260 nm.

#### 2.4.4. Swelling Rate

The swelling rate was determined to analyze the amount of water that can be stored inside the TCTS and conditioned TCTS hydrogels. In brief, the dry hydrogels were weighed (*Wd*) and then submerged in 10 mL of PBS for 2 h at 37°C. The samples were placed on nitrocellulose filter paper to eliminate the residual PBS and then weighed (*Ww*). The swelling ratio in percentage was determined using the following formula (Equation (1)):

(1) $$\begin{array}{c} \text{Swelling }\left(\%\right)=\left(\frac{Ww-Wd}{Wd}\right)\times 100. \end{array}$$

#### 2.4.5. Degradation Rate (Weight Loss Percentage)

Weight loss rate of the TCTS and conditioned TCTS hydrogels was examined by determining the weight loss of the samples after 35 consecutive days submerging in PBS. The samples were weighed (*W*1) submerged in 5 mL of PBS solution on a shaker inside the incubator at a temperature of 37°C. In consecutive time periods (Days 1, 7, 14, 21, 28, and 35), the samples were removed from PBS and completely dried, and then, the weight of the samples was measured (*W*2). The following formula was used to calculate the weight loss (percentage) of the hydrogels (Equation (2)):

(2) $$\begin{array}{c} \text{Degradation }\left(\text{weight loss}\%\right)\text{: }\left(\frac{W1-W2}{W1}\right)\times 100. \end{array}$$

### 2.5. SC/TC-Conditioned TCTS Interactions

#### 2.5.1. SC Isolation, Culture, and Expansion

Fifteen newborn NMRI male mice (3–6 days old) were used to harvest testicular tissue for isolation of SCs. After being separated from the animals, the testes were kept in sterile PBS until they were transferred to the culture medium for several washes with sterile PBS. SCs were isolated from the testes of newborn mice using two stages of enzymatic digestion and elimination of differentiation according to a protocol described by Asgari et al. [34].

#### 2.5.2. SC Adhesion to TC-Conditioned CTS Interactions

In order to study the effects of TC conditioning on the adhesion and morphology of SCs grown on the hydrogel scaffolds, 2 × 10^4^ spermatogonized cells were cultured on the surface of TCTS and TC-TCTS hydrogels. After 3 days of incubation in a standard cell culture incubator, the samples were prepared for photographing under SEM (Philips XL30). For this aim, a layer of gold was covered on the samples by sputtering method, and the samples were analyzed under SEM.

#### 2.5.3. SC Viability on TC-Conditioned CTS Interactions

To evaluate the effects of TC conditioning on the viability of SCs, 1 × 10^4^ cells were seeded on TCTS and TC-TCTS and incubated for 3 and 7 days in whole DMEM culture media at 37°C. After each interval incubation time, the cell/hydrogel construct was gently washed with sterile PBS, treated with whole DMEM contained with 10%MTT (Sigma, United States) solution (5 mg/mL) at 37°C for 4 h. The MTT solution was then gently discarded and replaced with dimethyl sulfoxide (DMSO, Sigma, United States). DMSO dissolves the formazan crystals formed in the mitochondria of the live cells, resulting in a purple solution. The optical density of the colored DMSO was measured at 570 nm using an ELISA microplate reader (DANA-DA3200, Iran). The cells cultured on the plastic surface of the cell culture plate served as a positive control (100% cell viability). The cell viability percentage was calculated using the following formula (Equation (3)):

(3) $$\begin{array}{c} \text{Cell viability}\left(\%\right)=\frac{\text{treated group cell number}–\text{average of negative control}}{\text{average cell number of positive control}}\times 100. \end{array}$$

### 2.6. Cell Proliferation–Associated Gene Expression Assay Using Real-Time PCR (q-RT-PCR)

5 × 10^5^ SCs were cultured on the TCTS and TC-TCTS hydrogels for 7 and 14 days. In the proliferation phase, 10 mg/mL GDNF and 10% KSR factors were used to improve the proliferation conditions. The cell culture medium was changed every 3 days. After 7 and 14 days of incubation, the cells were examined to investigate the expression of *id4* and *plzf* proliferation-associated genes using the q-RT-PCR method. For this aim, total RNA of the harvested cells was extracted using the Qiagen RNA extraction kit (Qiagen, Germany). The cDNA was synthesized by random hexamer and oligo dT primers using the cDNA synthesis kit (Qiagen, Germany). The expression of the targeted genes was analyzed and compared between TCTS and TC-TCTS using a q-RT-PCR RealTiQ 2x hot-start SYBR green master mix (Takara Shuzo, Japan) and the Rotor-Gene q-RT-PCR instrument (Qiagen, United States). The expression fold change was determined using the Log*ΔΔ*ct2 method [51]. In this analysis, the expression level of selected genes in the cell culture plate was considered as the control group (onefold change). GAPDH was used as a reference gene. The primers used in this study are listed in Table 1.

### 2.7. Statistical Analysis

Data analysis was done with GraphPad Prism 8 software, and graphs were drawn using SigmaPlot 12.3 software. The probability of significance (*p* ≤ 0.05) was considered a significant difference between the means. The ANOVA test was used to compare more than two groups, and the independent *t*-test was used to compare between two groups. The results of water absorption and weight loss rate were statistically analyzed with the independent *t*-test, and the results of DNA content, survival percentage (MTT), and expression of reproductive genes were analyzed with one-way ANOVA and the Tukey test. All experiments were performed in three replicates.

## 3. Results and Discussion

### 3.1. Characterization of CTS

#### 3.1.1. Inverted Tube Assay

**Figure 1:**
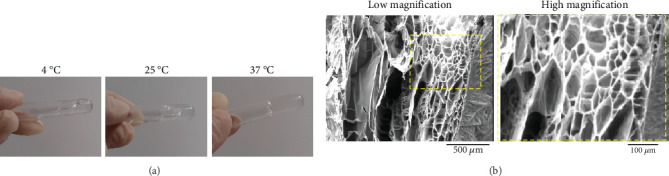
Characterization of thermosensitive chitosan hydrogel (TCTS). (a) Inverted tube test after incubation of TCTS for 15 min at 4°C, 25°C, and 37°C. TCTS remained in the solution phase at 4°C and 25°C, while it formed gel at 37°C. (b) The morphology of the TCTS hydrogel after the freeze drying process under SEM. The surface of the hydrogel had a porous, open, and interconnected structure.

In this study, the effect of temperature on the gelation of TCTS hydrogel was confirmed by the inverted tube method. As the temperature increased from 4°C to 37°C, the hydrogel turned into a gel, while at 4°C and 25°C the hydrogel was still in liquid form (Figure 1a). This result confirmed the thermoresponsive property of the fabricated TCTS hydrogel.

#### 3.1.2. Morphology Under SEM

The morphology of the freeze-dried TCTS in both high and low magnifications was observed under SEM (Figure 1b). The hydrogel showed a uniform microstructure with a smooth interconnected porous network. The pores were interconnected with sizes suitable for cell culture, cell proliferation and migration, oxygen/nutrient delivery, removal of waste, and protein transport.

### 3.2. Conditioning of CTS With TCs

#### 3.2.1. Isolation and Culture of TCs

**Figure 2:**
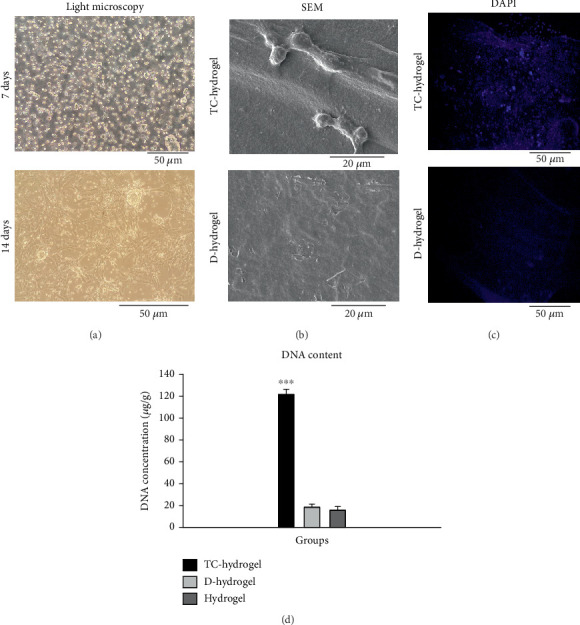
Fabrication of conditioned TCTS. (a) Examination of testicular cells extracted from rat testicular tissue after 7 and 14 days under light microscopy. After 7 days, cells were attached and few cells were detached. The cells on Day 14 have a uniform morphology. (b) Examining the surface of the TC-cultured hydrogel before and after decellularization under SEM. (c) The surface of the TC-TCTS before and after decellularization stained with DAPI under a fluorescence microscope. Cell's nuclei are stained in light blue fluorescence. (d) DNA content assay for TCTS (hydrogel), testicular cell-cultured TCTS (TC-hydrogel), and decellularized TCTS (D-hydrogel) samples. ⁣^∗∗∗^*p* < 0.001.

TCs extracted from mouse testis tissue were observed under a light microscope on Days 7 and 14 after extraction. After 14 days, the number of the cells increased and had a more uniform morphology with around 80% confluency (Figure 2a).

### 3.3. Characterization of TC-Conditioned TCTS

#### 3.3.1. Morphology Under SEM

The surface of hydrogel covered with ECM before and after decellularization was examined by SEM (Figure 2b). The TCs were well attached to the TCTS and grown actively on the substrate. After decellularization, the cells were removed from the TCTS substrate and left ECM components. The presence of the ECM that remained on the TCTS surface is clearly seen in Figure 2b. The scaffold before decellularization is smooth; however, the surface of hydrogel after decellularization is rough, indicating the presence of remained ECM or cell fragments.

#### 3.3.2. DAPI Staining

The presence of cells on the surface of hydrogel before decellularization and successful removal of the TC after decellularization was confirmed by DAPI staining (Figure 2c). In staining with DAPI, the cell nucleus or DNA fragments are stained with bright blue fluorescent color. As can be clearly seen, the cells' nuclei were stained with DAPI in cellular hydrogel, while no cell nucleus was observed in the decellularized hydrogel samples, indicating successful removal of the cells after decellularization.

#### 3.3.3. DNA Content Assay

By examining the amount of DNA content in the comparison of TC-cultured hydrogel scaffold samples (TC-hydrogel) with groups before culture with TCs (hydrogel) and after decellularization (D-hydrogel), it was observed that there was a significant difference between the genetic content between TC-hydrogel and hydrogel and D-hydrogel groups (*p* < 0.001). Also, by comparing the amount of DNA content between hydrogel and D-hydrogel groups, no significant difference was observed (Figure 2d). Along with SEM and DAPI staining, the DNA content assay confirmed the decellularization.

#### 3.3.4. Swelling Rate

**Figure 3:**
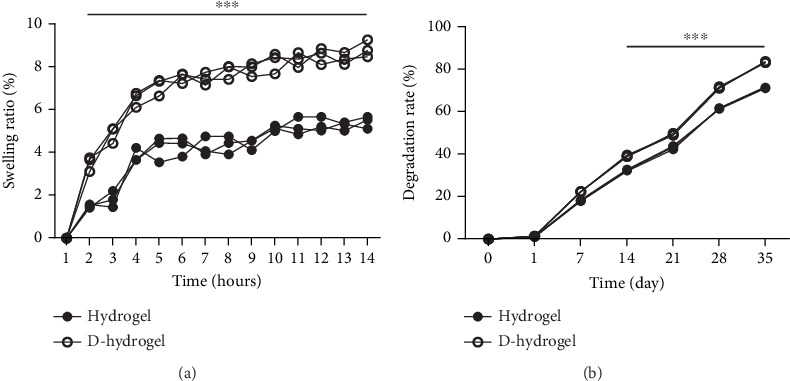
Swelling rate and biodegradation (weight loss percentage) assays. (a) Volume percentage of water absorption by temperature-sensitive chitosan scaffold (hydrogel) and chitosan hydrogel containing extracellular matrix (D-hydrogel) during 14 days of follow-up. ∗∗∗ indicates a significant difference between hydrogel and D-hydrogel groups in each time is separate. (b) Weight loss percentage of chitosan temperature-sensitive scaffold (hydrogel) and hydrogel containing extracellular matrix (D-hydrogel) during Days 1, 7, 14, 21, 28, and 35. ∗∗∗ indicates a significant difference between hydrogel and D-hydrogel groups at each time point separately.

The percentage of water absorption (swelling ratio percentage) by TCTS before (hydrogel) and after (D-hydrogel) conditioning with TC ECM within 14 h is shown in Figure 3a. On the 14th hour, the hydrogel and D-hydrogel groups absorbed water around 56% and 87% of their initial weight, respectively. Conditioning significantly increased the swelling rate of the hydrogels. The significant differences began after 2 h submerging in PBS at 37°C.

#### 3.3.5. Degradation Rate (Weight Loss Percentage)

The weight loss of TCTS and D-TCTS is examined and presented in Figure 3b. The weight loss percentage in TCTS and D-TCTS after 35 days submerging in PBS was about 69% and 82%, respectively. The significant differences in losing weight began after 14 days submerging in PBS at 37°C.

### 3.4. SC/TC-Conditioned TCTS Interactions

#### 3.4.1. SC Isolation and Culture

**Figure 4:**
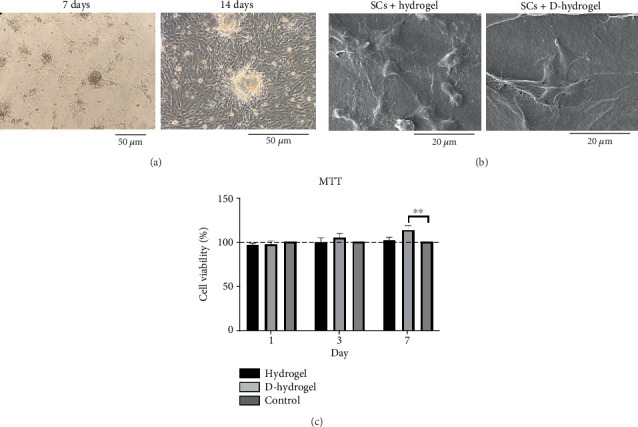
Examining the extracted spermatogonia cells (SCs) on the scaffolds. (a) Spermatogonia cells did not have a uniform appearance after 7 days. The cells showed a uniform morphology after 14 days with a uniform colony formation. (b) Morphology of SCs on TCTS hydrogel scaffold without extracellular matrix (hydrogel) and conditioned with extracellular matrix (D-hydrogel). The cells on the matrix hydrogel are much more elongated and had a greater tendency to adhere to the scaffold surface. (c) Viability of SCs on hydrogel and D-hydrogel during 1, 3, and 7 days of cultivation was examined by MTT test. ∗∗ indicates significant difference with control (*p* ≤ 0.05).

The morphology of the SCs isolated and expanded in the cell culture plate at Days 7 and 14 postisolation is shown in Figure 4a. After 14 days, the number of the cells increased and had a more uniform morphology and colony with around 80% confluency.

#### 3.4.2. SC Adhesion to TC-Conditioned TCTS Interactions

The morphology of SCs on the TCTS (hydrogel) and TCTS conditioned with TC ECM (D-hydrogel) was investigated under SEM (Figure 4b). SCs were well attached on both hydrogels, but as can be seen, these cells on the D-hydrogel had much more elongated morphology and were more inclined to stick to the hydrogel surface covered with ECM. This observation confirms the positive effect of TC conditioning on the cell adhesion property of the TC-TCTS.

#### 3.4.3. SC Viability on TC-Conditioned TCTS Interactions

The survival percentage of SCs on the hydrogel without ECM (hydrogel) and conditioned with ECM (D-hydrogel) is shown in Figure 4c. On the 7th day, a significant increase in the survival percentage of SCs on the D-hydrogel scaffold compared to the control and hydrogel groups was observed. The percentage of survival of the hydrogel group was 101.69%, 99.62%, and 96.30% on Days 1, 3, and 7, respectively. The survival rate of the D-hydrogel group on Days 1, 3, and 7 was 113.57%, 105.31%, and 97.25%, respectively. All the samples showed cytobiocompatibility for SCs during the 7 days of follow-up.

### 3.5. Proliferation Gene Expression Assay Using q-RT-PCR

**Figure 5:**
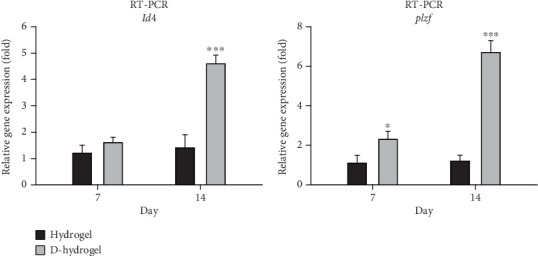
Gene expression evaluations by q-RT-PCR. Comparison of the expression of *id4* and *plzf* proliferation genes in spermatogonia cells on Days 7 and 14 after cultivation on hydrogel without extracellular matrix (hydrogel) and conditioned with TC extracellular matrix (D-hydrogel) compared to the group (the cells cultured on cell culture plate) of cultured cells.

The expression of *id4* and *plzf* genes in SCs during 7 and 14 days of cultivation on TCTS (hydrogel) and TC-conditioned TCTS (D-hydrogel) was evaluated and compared by q-RT-PCR method. As shown in the diagram of Figure 5, no significant difference was seen between the *id4* gene expressions among the groups on Day 7, but on Day 14 of cell culture, the D-hydrogel group caused a significant increase in the expression of this proliferation gene in SCs compared to the control group and hydrogel (*p* < 0.001). Also, the D-hydrogel group showed a significant increase in *plzf* gene expression compared to the hydrogel and control groups at both 7 (*p* ≤ 0.05) and 14 (*p* < 0.001) days of culture.

## 4. Discussion

Biological environment plays a pivotal role in the growth and regeneration of neo-tissue. Each tissue has a specific niche, provided by ECM components, playing an instructive role for a variety of cellular activities and providing bioactive cues for proliferation and differentiation, particularly germ cell differentiation [25, 52–55].

In the present study, a microenvironment was fabricated from chitosan in thermosensitive hydrogel form and then decorated with specific ECM secreted from TCs to biomimic natural testicular tissue and facilitate the proliferation, growth, and differentiation of sperm-producing cells. To this aim, chitosan hydrogel was treated with an optimized concentration of *β*-glycerophosphate crosslinker to form a layer of hydrogel film on the plastic surface of cell culture plate at 37°C and provide a 3D biomimicking substrate for cell culture. The effect of temperature on the gelation of temperature-sensitive chitosan hydrogel was confirmed by the inverted tube method. According to the results obtained by increasing the temperature from 4°C to 37°C, the hydrogel turned into a gel, so that at 4°C and 25°C, the hydrogel was still in liquid form. *β*-Glycerophosphate modulates CTS chains' hydrogen bonding and hydrophobic and electrostatic interactions [56]. Hydrogels sensitive to temperature and pH have special applications in tissue engineering and biomaterials compared to other types of hydrogels sensitive to the environment [57, 58]. For example, hydrogels sensitive to the environment are used for the application of drug release according to external stimuli such as pH or temperature [59]. The first report on chitosan hydrogels was recorded by Chenite and his colleagues in 2000. Their study showed that the free amino groups in the chitosan chain are protonated at low pH and lead to electrostatic repulsion between the polymer chains and finally the liquid solution structure. Their results showed that a pH above 6.2 leads to the neutralization of chitosan chains and accelerates the gelation process. They found that chitosan–beta-glycerol phosphate solutions remain liquid at < 25°C, even with pH values ranging from 6.8 to 7.2. This system becomes sensitive to temperature, which is liquid at < 25°C and turns into a gel by increasing the temperature to body temperature [60]. The results of examining the structure morphology of chitosan temperature-sensitive hydrogel scaffold with electron microscope showed a porous structure with interconnected pores. Although studies have shown that observing the microstructure of hydrogels after losing their water cannot give us complete information about the microstructure, some studies showed that the structure of the hydrogel may depend on the type of acid used in the preparation of the chitosan solution. For example, the use of acetic acid creates a porous, open, and interconnected structure. In contrast, hydrogels prepared with propionic acid or lactic acid have a scaly porous structure and very large pore diameters [61–63]. Therefore, the resulting structure can be caused by the use of acetic acid in the production of hydrogel. Our samples showed a porous structure with internal joints ranging from 100 to 300 *μ*m. The results of our study showed that the walls of the pores, which are branched, indicate the creation of suitable surfaces for cell connections [64, 65].

Today, the use of natural scaffolds derived from decellularized tissues has received the special attention of researchers because, in these scaffolds, cell antigens are removed by the decellularization process, but many structural and functional proteins of the ECM are preserved [66]. Many studies have reported that the ECM plays an important role in inducing the differentiation of stem cells into different cells [67, 68]. As described earlier, in the present study, the TCTS hydrogel scaffold was recellularized with TC for 14 days and then decellularized to finally make a scaffold with ECM of TCs. Examining the morphology of the hydrogel with ECM compared to the hydrogel without ECM using SEM confirmed that the surface of the scaffold was covered with matrix. The results of staining both hydrogels with ECM and without ECM with DAPI showed that the resulting scaffold did not contain TCs. The freeze and thawing caused the rupture of the cells grown on the hydrogel and successful removal of the cells during the freeze-thawing decellularization method. The results obtained from the investigations of water absorption and weight loss of the hydrogel scaffold showed a significant increase in the amount of water absorption and also acceleration of weight loss after being covered with ECM. The rate of water absorption in the first 4 h had a high speed, and from 5 to 14 h, there was a slower upward trend. As observed, conditioning with ECM caused a significant increase in water absorption within 24 h so that the hydrogel before and after being covered with ECM had water absorption equal to 56% and 87% of their initial weight, respectively. Degradation of chitosan/beta-glycerol phosphate hydrogel was studied in saline buffer environment after conditioning with ECM of TCs at 37°C. The weight loss (percentage) of the sample started at a significant speed at first. Then, the rate of weight loss for the sample decreased. Also, the rate of weight loss of the hydrogel after the placement of the ECM showed a significantly faster rate than the hydrogel without the matrix from Day 7 onwards, and this increase in the percentage of weight loss continued until 35 days in the same process. After 35 days, the hydrogel without ECM (hydrogel) and with ECM (D-hydrogel) lost about 69% and 82% of their weight, respectively. A possible explanation for the water absorption and swelling result could be the hydrophilic nature of ECM attached to the hydrogel microenvironment. In this way, increasing the hydrophilicity of the hydrogel increased the penetration of water into the pores (absorption of water) and also increased the rate of weight loss due to it [69, 70]. Also, in this study, the measurement of the DNA content was used to confirm the complete decellularization of the hydrogel scaffold. Our results showed that the amount of DNA in hydrogels after decellularization significantly reduced compared to recellularized hydrogels. There was a significant difference between the genetic content after culturing with TCs (TC-hydrogel) and the groups before culturing with TCs (hydrogel) and after decellularization (D-hydrogel). Also, by comparing the amount of DNA content between the hydrogel and D-hydrogel groups, no significant difference was observed [71].

In order to check the viability of SCs, the MTT test was used in the ECM hydrogel scaffold of TCs. The results showed that there was no significant change in the viability of SCs on Days 1 and 3 between the hydrogel and control groups (cells cultured on the plastic surface of cell culture). But on Day 7, there was a slight but significant difference for SCs on D-hydrogel compared to other groups. Live cells with active metabolism convert MTT to a purple formazan product with an absorption maximum near 570 nm. Therefore, the intensity of the colored product is directly proportional to the number of living cells in the culture [72]. Observation of SCs with SEM showed that SCs did not have a uniform appearance after 7 days. But after 14 days, they had a uniform morphology and formed a colony. The cells cultured on the conditioned hydrogels showed noticeably more elongated than the cells cultured on the hydrogel scaffold without ECM. These results confirm a remarkable increase in the biocompatibility and adhesion of the scaffold after being covered with the ECM of TCs. A similar study in order to cover the ECM of osteoblast cells on a 3D gelatin/hydroxyapatite scaffold for bone tissue engineering was conducted by Samadikuchaksaraei and his colleagues in 2016. Their results showed that conditioning the surface of the scaffold with the matrix of osteoblast cells had a great effect on increasing the cell adhesion of the scaffold as well as repairing bone lesions in an in vivo study [73]. The results of investigating the effect of the hydrogel scaffold with ECM on the expression of premeiotic proliferation genes (*id4* and *plzf*) in SCs by the q-RT-PCR method showed that the presence of ECM increased the expression of these genes in SCs cultured on the scaffold after 7 and 14 days. There was no significant difference in the id4 gene expression between the groups on the seventh day, but on the 14th day of cell culture, the D-hydrogel group caused a significant increase in the expression of this gene in SSCs compared to the hydrogel group. Also, the D-hydrogel group showed a significant increase in *plzf* gene expression compared to the hydrogel group at both 7 and 14 days of culture time. Talebi et al. [74] cultured the SCs isolated from newborn mice on polycaprolactone scaffolds covered by a soft agar layer for 2 weeks. Their results showed that the colony formation rate of SCs was significantly induced in the modified soft agar culture system. The expression level of Id4, *Plzf*, and *Gfrα1* genes was higher in the 3D culture system compared with the control group. In addition, the expression of the *c-kit* gene, as a differentiating marker of spermatogonia, was higher compared to the values of other experimental groups in the presence of the scaffold and soft agar. As a result, soft agar induced the expression of *Id4* and *plzf* genes and downregulated the expression of the *c-kit* gene. The results of their study show a positive correlation between the proliferation of SCs and culture in soft agar medium.

The main aim of our study was to investigate the potential of hydrogel scaffold with ECM of cells controlling the proliferation of SCs, as a system for the culture and proliferation of SC stem cells. The results of this study suggest the hydrogel scaffold conditioned with the ECM of TCs as a candidate with high potential for proliferation, growth, and adhesion of SCs, as well as a system for sperm production. Nevertheless, further investigations should be conducted in the next phase of this study to evaluate the production of functional sperms on this engineered construct.

## 5. Conclusion

In this study, a temperature-sensitive hydrogel scaffold made of chitosan conditioned with the ECM of TCs was designed in the hope that, in the future, it will provide a suitable system for the growth and proliferation of SCs and sperm production in a laboratory environment. Therefore, the design and construction of a 3D bed to imitate the natural tissue of spermatogenic tubes can be promising for the production of spermatogenic cells or efficient sperm in the laboratory environment. Of course, additional studies and more tests are needed in order to finally confirm the differentiation and production of sperm by this method and to check the health of the produced sperm, which will be examined in the next study.

## Figures and Tables

**Table 1:** Primers used in the current study.

**Gene**	**Size (base pair)**	**Sequence (5**⁣′** to 3**⁣′**)**	**Annealing temperature (°C)**
*Plzf*	137	F: CCCGTTGGGGGTCAGCTAGA R: CTGCAAGGTGGGGCGGTGTAG	61
*Id4*	185	F: GGGTGACAGCATTCTCTGC R: TTGGAATGACAAGACGAGACG	58
*GAPDH*	125	F: CTGCTGGACAAGTGAGTCCC R: CCAAGTACCCTGGCCTCATC	60

## Data Availability

The data that support the findings of this study are available from the corresponding author upon reasonable request.
